# The Potential Teeth Bleaching and Halitosis Prevention Effects of *Pediococcus inopinatus* THK-30, a Kimchi-Derived Lactic Acid Bacterium: In Vitro Study

**DOI:** 10.3390/jfb15030064

**Published:** 2024-03-07

**Authors:** Trang Thi Minh Nguyen, Qiwen Zheng, Eun-Ji Yi, Arce Defeo Bellere, Xiangji Jin, Hong-Yong Kim, Tae-Hoo Yi

**Affiliations:** 1 Graduate School of Biotechnology, Kyung Hee University, Yongin 17104, Republic of Korea; trangnguyen@khu.ac.kr (T.T.M.N.); zhengqiwen@khu.ac.kr (Q.Z.); 0201@khu.ac.kr (E.-J.Y.); arcedbellere@khu.ac.kr (A.D.B.); junoenc73@khu.ac.kr (H.-Y.K.); 2 Snowwhitefactory Co., Ltd., 807, Nonhyeon-ro, Gangnam-gu, Seoul 06032, Republic of Korea; 3 Department of Dermatology, School of Medicine, Graduate School, Kyung Hee University, 26 Kyungheedae-ro, Dong-daemun, Seoul 02447, Republic of Korea; hyanghe112@khu.ac.kr; 4 J1 Cosbio Co., Ltd., 820, Seonyu-ro 13-gil 25, Yeongdeungpo-gu, Seoul 07282, Republic of Korea

**Keywords:** *Pediococcus inopinatus*, sodium hexametaphosphate, teeth bleaching, antibacterial, halitosis, complementary and alternative medicine

## Abstract

Background: Recent developments in addressing dental aesthetic concerns, encompassing issues like teeth discoloration and halitosis, underscore the demand for safer alternative solutions. Purpose: This study aims to confirm the effects of lactic acid bacteria (LAB) from kimchi on artificial teeth bleaching and their potential impact in terms of preventing halitosis-related bacteria. Materials and Methods: To evaluate the antimicrobial effects against oral pathogens, disc diffusion tests and broth microdilution methods were used. Additionally, crystal violet analysis was performed to confirm the biofilm inhibition effect. The bleaching effects on stained artificial teeth were analyzed using the CIEDE2000 colorimetric method. Statistical analyses were performed using GraphPad Prism 9 with one-way and two-way ANOVA, with the significance level set at α < 0.05. Results: The strain THK-30, isolated from kimchi, exhibited antibacterial activity against *Streptococcus mutans*, *Porphyromonas gingivalis*, and *Fusobacterium nucleatum*, and was identified as *Pediococcus inopinatus*. Moreover, THK-30 showed a synergistic antibacterial effect against Gram-negative oral pathogens with 8% sodium hexametaphosphate (SHMP). In the stained artificial teeth bleaching test and artificial teeth biofilm inhibition test, the cell-free supernatant of THK-30 displayed significant teeth bleaching effects and caused the inhibition of biofilm formation, both independently and in combination with SHMP 8%. Conclusions: This study has demonstrated the potential applicability of LAB in teeth discoloration and halitosis. These findings are poised to provide a foundation for the development of research pertaining to the control of oral bacteria.

## 1. Introduction

Recent advancements in medicine and science have led to a shift in humanity’s focus from mere lifespan extension to enhancing the quality of life. This paradigm shift has permeated various aspects of healthcare, including the field of dentistry [1]. With an increasing emphasis on improving overall well-being, there has been a notable transition from solely addressing dental diseases to meeting aesthetic demands as well [2]. Specifically, there is growing attention being paid towards addressing halitosis [3] and bleaching [4], indicating a broader spectrum of concerns within oral health care.

The discoloration of teeth, commonly referred to as bleaching, arises from a multitude of factors, including enamel erosion, dentin exposure, trauma, plaque accumulation, and lifestyle habits such as diet and smoking [5]. Notably, the consumption of staining substances like coffee, tea, and tobacco exacerbates this issue, affecting both natural and artificial teeth [6,7]. As procedures involving artificial teeth, such as implants, crowns, and bridges, witness a surge in popularity, there arises a pressing need to explore solutions for artificial teeth discoloration, an area that has been relatively understudied compared to natural teeth bleaching [8].

**Figure 1:**
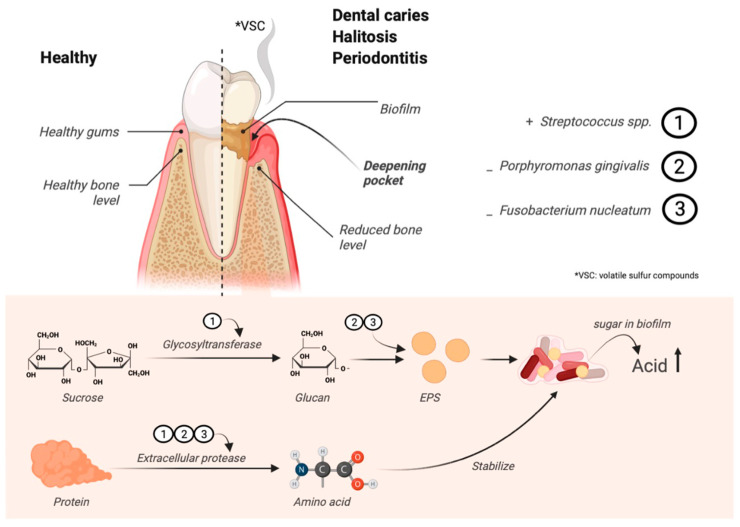
Biofilm-forming mechanism of oral pathogens. The figure was generated by BioRender.com.

Halitosis, commonly known as bad breath, stems from volatile sulfur compounds (VSC) produced by bacteria within the oral cavity [9]. Its prevalence among the global adult population, estimated at 20–50%, underscores its significance as a social and psychological concern [10]. The causes of halitosis are multifaceted, encompassing gastrointestinal disorders and oral bacterial overgrowth [11], with oral bacteria, particularly those residing within dental plaque, identified as primary culprits [12]. The detailed mechanisms of this biofilm formation center on two key enzymes, glycosyltransferase and protease, responsible for creating biofilm matrices, including exopolysaccharides (Figure 1). 

The effective management of oral bacteria and plaque inhibition are paramount in both bleaching and halitosis control [13,14]. While current methods such as brushing, hydrogen peroxide application, and chemical mouthwash exist, their efficacy and sustainability remain questionable due to issues related to toxicity and their prolonged effects [15,16]. Hence, there is a compelling need for the development of safe and efficacious strategies for use in bleaching and halitosis management.

Kimchi, a traditional Korean fermented food made from ingredients such as cabbage, radish, and garlic, has gained recognition, not only for its rich flavor but also for its diverse health benefits [17]. It is believed to possess various health-promoting functions, preventing androgenic alopecia [18], performing immunomodulatory functions [19], and managing weight [20]. Kimchi stands out as an excellent example of utilizing LAB as a starter organism, with numerous articles focusing on its isolation [21]. Various studies have explored the antibacterial, anti-adipogenic, antioxidant, and cholesterol-controlling effects of LAB derived from kimchi [17].

*Pediococcus inopinatus* (*P. inopinatus*) is a strain of LAB found naturally in fermented food products like kimchi [22]. *P. inopinatus* has been studied for its effectiveness in relieving hangovers, as well as its antibacterial and anti-allergy effects, further suggesting its applicability in the food industry [23,24,25].

The aim of this study was to investigate the potential of *P. inopinatus* derived from kimchi for use in teeth bleaching and the treatment of oral bacterial infections that cause halitosis. The findings of this study are expected to have the potential to significantly enhance the development of effective and safe treatments for dental aesthetics and oral health issues.

## 2. Materials and Methods

### 2.1. Bacteria Isolation, Identification, and Cultivation

The kimchi soup underwent serial dilution in a 0.85% NaCl solution to achieve concentrations ranging from 10^−6^ to 10^−9^. Subsequently, it was spread on bromocresol purple (BCP; Eiken Chemical Co., Ltd., Tokyo, Japan) agar and incubated at 30 °C for two days. Colonies displaying yellowish rings, characteristic of LAB pH changes, were selectively isolated [25]. These colonies were transferred to De Man, Rogosa, and Sharpe (MRS) broth and agar (Difco, Detroit, MI, USA) for routine culturing at 30 °C. To determine strain homology, this paper used the 16S rRNA sequence technique with primers 27F and 1492R. The resulting sequence underwent analysis, using the EzBioCloud database as a sequence analysis tool (accessed on 15 November 2022). Biochemical analysis, particularly the carbohydrate fermentation test, was conducted using an analytical profile index of 50 CH kits (API; BioMérieux, Marcy l’Etoile, France).

### 2.2. Bacterial Growth Conditions and Metabolite Preparation

The oral pathogenic strains were purchased from the Korean Collection for Type Cultures (KCTC) and the Korean Agricultural Culture Collection (KACC). The strains used in this study were *Streptococcus mutans* (*S. mutans*) KACC 16833, *Streptococcus gordonii* KACC 13829, *Streptococcus mitis* KACC 16832, *Streptococcus downei* KACC 13827, *Streptococcus ferus* KACC 13881, *P. gingivalis* KCTC 5352, and *F. nucleatum* KCTC 2640. Tryptic soy broth with 0.3% yeast extract (*w*/*v*) (TSY; OXOID, Basingstoke, UK) was used under aerobic conditions at 30 °C for *Streptococcus* spp., while reinforced clostridial broth (RCM; OXOID, Basingstoke, UK) media were used under anaerobic conditions at 30 °C for Gram-negative strains. LAB selected for the study was grown in modified MRS broth with 2.0% glucose, 2.5% yeast extract, 0.5% sodium acetate, 0.1% polysorbate 80, 0.2% potassium phosphate, 0.2% ammonium citrate, 0.01% magnesium sulfate, and 0.005% manganese sulfate at 30 °C for 48 h to produce mass metabolites used for artificial-teeth-related experiments, while commercial MRS media were used to produce a cell-free supernatant (CFS) for other tests included in the paper. The culture medium was subjected to centrifugation at 12,000 rpm for 10 min, after which the resulting supernatant was filtered through a 0.22 μm membrane filter. The resulting filtrates were employed directly as samples to screen antibacterial activity. Additionally, the filtrates that were concentrated using a centrifugal evaporator (EYELA, Tokyo, Japan) at 45 °C were used for further assessment of antibacterial and antibiofilm activity.

### 2.3. Teeth Bleaching Test Preparation

#### 2.3.1. Artificial Teeth Staining

Vipi A3 resin denture teeth (Vipi, Pirassununga, Brazil) were utilized in the staining tooth preparation phase. These teeth were chosen for their high color stability and realistic appearance. A total of 128 teeth were used in the experiment, with 3 teeth for each subgroup. After delicately scraping off the top wax coating of the artificial teeth using sandpaper (number #150), the teeth were then stained using a black tea (Lipton yellow label tea, Lipton, Istanbul, Turkey) solution. The tea solution (one bag per 100 mL water) was changed every 24 h during the ten-day staining process.

#### 2.3.2. Teeth Bleaching Assay

The study included two groups for the teeth bleaching test [26,27]. The brushing and mouthwash group was placed in a wax tray and dipped for 1 s into the sample solution, brushed for 2 min, and then dipped once more. With the in-office bleaching group, each tooth was submerged in each sample solution for 40 min. After treatment, teeth were cleansed with a paper towel to remove the residual sample solution, and the teeth were stored in 37 °C conditions in a 15 mL conical tube with 2 mL of sterile distilled water.

Colorimetric measurements of treated tooth surfaces were conducted using the digital spectrophotometer Vita Easy Shade Advance 4.0 (VITA Zahnfabrik, Bad Säckingen, Germany) [28]. The Whiteness Index for Dentistry (WI_D_), a whitening index based on the CIE L*a*b* color space, is utilized in dentistry to assess the relationships among perceptions of tooth color. WI_D_ effectively addresses the limitations observed in previous whiteness indices through validation experiments. The calculations were performed using the following formula:

WI_D_ = 0.511L* − 2.324a* + 1.100b*

In this equation, the L* values, ranging from 0 (representing black) to 100 (indicating white), signify the lightness of color. The a* values, where −a denotes greenness and +a signifies redness, highlight the color distinctions between green and red. Simultaneously, the b* values, with −b representing blueness and +b indicating yellowness, signify the differences between blue and yellow coordinates. The outcomes derived from the provided formula demonstrate that higher WI_D_ values correspond to whiter samples, while lower WI_D_ values, including negative values, suggest darker samples. These measurements were carried out both before (baseline) and after the application of the bleaching agent.

In the formulation of the color difference metric CIEDE2000, the ΔE_00_ color difference formula is used to quantify the perceived change in tooth color before and after the bleaching process. This formula considers variations in lightness (ΔL*), chroma (ΔC*), and hue (ΔH*), with factors and adjustments weighted to align with human visual perception. Specific parametric values are assigned to lightness (*K*_L_), chroma (*K*_C_), and hue (*K*_H_). Visual–instrumental color harmony within dentistry requires that parameters of *K*_L_ = 2, *K*_C_ = 1, and *K*_H_ = 1 be adopted, adhering to a differentiated weighting scheme of 2:1:1 [29]. The rotation term (RT) addresses interactions between chroma and hue [30]. The ΔE_00_ color difference formula is represented as follows:

ΔE_00_ = [(ΔL*/*K*_L_*S*_L_)^2^ + (ΔC’/*K*_C_*S*_C_)^2^ + (ΔH’/*K*_H_*S*_H_)^2^ + *R*_T_(ΔC’/*K*_C_*S*_C_) (ΔH’/*K*_H_*S*_H_)]^1/2^

The perceptibility threshold (PT), denoted as 50:50%, signifies the point at which 50% of observers can discern a color difference. The acceptability threshold (AT), also denoted as 50:50%, indicates the level at which 50% of observers find the difference acceptable. In an investigation utilizing CIEDE2000, the 50:50% PT was acknowledged at ΔE_00_ = 0.8, while the corresponding 50:50% AT was determined to be ΔE_00_ = 1.8 under simulated clinical conditions.

### 2.4. Broth Microdilution Method

The study employed the broth microdilution assay recommended by the guidelines of the Clinical and Laboratory Standards Institute to ascertain the minimum inhibitory concentration (MIC) and minimum bactericidal concentration (MBC) of the chosen LAB [31]. The evaporated CFS of the selected LAB was serially diluted and inoculated into a 96-well microtiter plate (Thermo Fisher Scientific, Waltham, MA, USA), with 100 μL in each well. Then, each well was supplemented with 100 μL of oral pathogenic strains in the TSY broth (1 × 10^6^ CFU/mL) and incubated at 30 °C for 24 h. The optical density (OD) was assessed at 595 nm, and the concentration exhibiting an OD of either 20% or less than that of the control group was designated as the MIC using a microplate reader (Molecular Devices, San Francisco, CA, USA). Next, a fraction of each well was streaked onto a TSY agar plate using a 1 μL inoculation loop, and the plates were incubated at 30 °C for 24 h to count the number of colonies. The concentration of LAB that resulted in the complete inhibition of colony formation on TSY agar plates was identified as the MBC. These techniques were utilized to evaluate the MIC and MBC of selected LAB against oral pathogenic strains in TSY broth for Gram-positive pathogens and RCM broth for Gram-negative pathogens.

### 2.5. Biofilm Formation Crystal Violet Assay

To assess the effect of CFS-THK-30 on biofilm formation, various concentrations of CFS-THK-30 (0, 0.08, 0.31, 1.25, 5, 10, 20, and 40 mg/mL) were added to 96-well microtiter plates already containing 100 μL dilutions of oral pathogenic strains (1 × 10^6^ CFU/mL), and these were incubated without shaking for 24 h at 37 °C [32]. After incubation, the solution was removed, and the wells were washed twice with phosphate-buffered saline (PBS). Afterward, 100 μL of 0.01% crystal violet solution (in 0.1% acetic acid) was added to each well and incubated for 15 min, followed by washing twice with PBS and air-drying. The biofilm-forming inhibition was evaluated by releasing the fixed crystal violet with 33% acetic acid and measuring the absorbance at 595 nm using a microplate reader. As part of the biofilm formation test, MRS broth that had been evaporated was employed as a control.

### 2.6. SHMP and THK-30 Synergy Test

The double-disc synergy test was performed for this test on TSY and RCM agar for Gram-positive and -negative strains, respectively. Discs containing antibacterial samples were placed 5–7 mm apart on a lawn culture of the target pathogens. Following incubation at 30 °C, the appearance of synergy was indicated by an increase in the zone diameter of more than 2 mm compared to that of the single agent or by the merger of the zones of inhibition [32]. A zone diameter increase of less than 2 mm was classified as weak synergy. Simultaneously, antagonism was demonstrated by cutting short the zone of inhibition at the intersection of the two antibacterials. Then, MIC and MBC assays were performed on the mixture of SHMP and THK-30 via the broth microdilution method.

### 2.7. Teeth Biofilm Assay

The artificial teeth were used as a surface base for biofilm formation, with the top coating scraped off using sandpaper [33]. These teeth were placed in 15 mL conical tubes. In each tube, a 3 mL bacterial suspension containing *S. mutans* was added, resulting in a final concentration of McFarland 3. This was approximately 9 × 10^8^ CFU/mL in TSYS media. After 24 h at 30 °C, the teeth were rinsed twice with distilled water to remove non-adherent bacteria. The biofilm on the teeth was then stained with 0.01% crystal violet for 20 min at room temperature. The excess stain was removed twice with distilled water. To measure the biofilm biomass on the teeth, the remaining crystal violet was extracted using 33% acetic acid for 2 h in shaking conditions. Biofilm biomass was quantified by measuring absorbance at OD_595_.

### 2.8. Enzymatic Characterization of Sample CFS

Three distinct treatment groups of sample CFS were examined [34]. For the pH stability test, a pH adjustment to 6.5 was performed on sample CFS to neutralize acidity with 0.1 M NaOH or 0.1 M HCl. The second treatment was pH adjustment, followed by catalase treatment at 0.5 mg/mL at 30 °C for 1 h to achieve the effect of catalase. The next treatment was pH adjustment, followed by digestion at 55 °C for 1 h with 0.1 mg/mL proteinase K. Subsequently, proteinase K activity was inactivated at 65 °C for 15 min to identify the effect of proteinase K on antibacterial substances in CFS. MRS broth, with the same treatment in each treatment group, was used as a control. All samples were diluted to the MIC concentration using the broth microdilution method, and these experiments were conducted in triplicate. OD_595_ was measured at 0, 6, 12, 18, and 24 h time.

### 2.9. Statistical Analysis

The statistical software GraphPad Prism 9, developed by GraphPad Software Inc. in La Jolla, CA, USA, was employed to perform both one-way ANOVA and two-way ANOVA analyses. The data were gathered in three separate replications, and the mean ± standard deviation was presented. A significance level of *α* < 0.05 and *p* < 0.01 were used for statistical evaluations for the teeth bleaching experiment and the remaining experiments, correspondingly.

## 3. Results

### 3.1. Isolation of Antibacterial LAB

**Figure 2:**
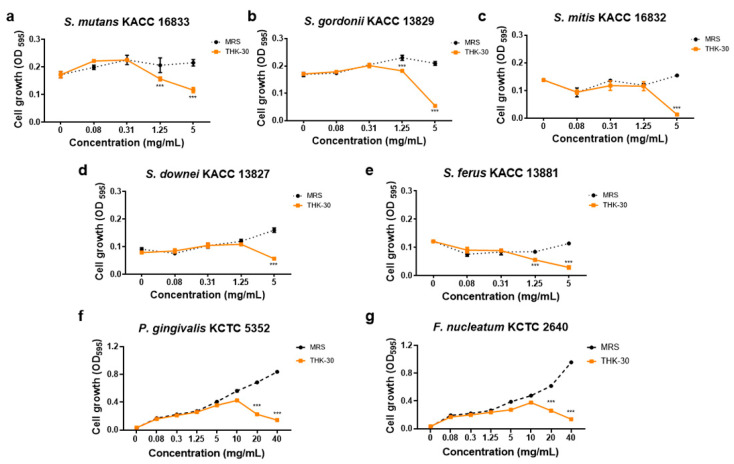
Antibacterial activity by CFS of *Pediococcus inopinatus* THK-30 against oral pathogens. (**a**) *Streptococcus mutans* KACC 16833; (**b**) *Streptococcus gordonii* KACC 13829; (**c**) *Streptococcus mitis* KACC 16832; (**d**) *Streptococcus downei* KACC 13827; (**e**) *Streptococcus ferus* KACC 13881; (**f**) *Porphyromonas gingivalis* KCTC 5352; and (**g**) *Fusobacterium nucleatum* KCTC 2640. All experiments were performed at least three times, and the data are presented as mean ± standard deviation. *** *p* < 0.001 vs. control group.

Forty strains of LAB were obtained from kimchi using the BCP plate pH color-changing characteristic. Within the selected candidates, strain THK-30 demonstrated significant antibacterial activity against oral pathogen bacteria after screening the MIC and MBC values. The results showed that THK-30 CFS inhibited the growth of all tested *Streptococcus* spp. strains, with *Streptococcus ferus* KACC 13881 showing a significantly stronger inhibition effect with MIC and MBC values of 1.25 and 2.5 mg/mL, respectively (Figure 2 and Table 1). With regards to Gram-negative pathogens, the MIC and MBC values of THK-30 CFS against *P. gingivalis* KCTC 5352 and *F. nucleatum* KCTC 2640 were detected and found to be effectively inhibiting from the concentration of 20 mg/mL above, as presented in Table 1 and Figure 2.

### 3.2. Identification of Strain THK-30

**Figure 3:**
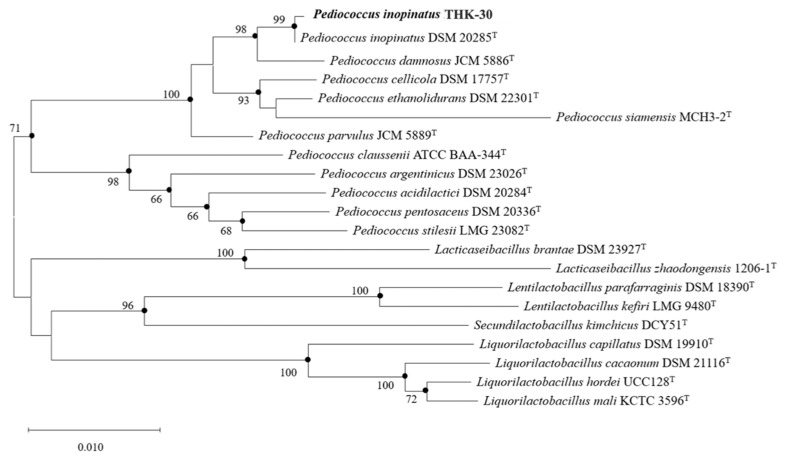
Neighbor-joining phylogenetic tree of strain THK-30. Bootstrap values (expressed as a percentage of 1000 replications) > 65% are shown at the branch points. Bar indicates 0.01 substitutions per nucleotide position.

The phylogenetic analysis of the 16s rRNA gene on strain THK-30 revealed its closest relative to be *Pediococcus inopinatus* DSM 20285^T^, with a similarity of 99.93%. This relationship was further confirmed by the phylogenetic tree displayed in Figure 3. The biochemical and cultural characteristics of strain THK-30 were compared with those of *Pediococcus inopinatus* DSM 20285^T^, the strain exhibiting the highest similarity identified in the phylogenetic analysis presented in Table 2. Both strains were observed to grow at a range of temperatures from 25 to 45 °C, with the most suitable temperature being 30 °C. The API 50 CH tests showed that all strains exhibited positive acid production for D-galactose, D-glucose, D-fructose, D-mannose, N-acetylglucosamine, esculin ferric citrate, salicin, D-cellobiose, D-trehalose, and gentiobiose. Conversely, all strains displayed negative acid production for glycerol, erythritol, D-arabinose, L-arabinose, D-ribose, D-xylose, L-xylose, methyl-β-D-xylopyranoside, L-sorbose, L-rhamnose, dulcitol, inositol, D-mannitol, D-sorbitol, methyl-α-D-glucopyranoside, arbutin, D-lactose, D-melibiose, D-saccharose, inulin, D-melezitose, D-raffinose, amidon, glycogen, xylitol, D-lyxose, D-tagatose, D-fucose, L-fucose, D-arabitol, L-arabitol, potassium gluconate, potassium 2-ketogluconate, and potassium 5-ketogluconate. The results indicate that the strain THK-30 is closely related to *P. inopinatus* DSM 20285^T^ and shares several biochemical and cultural characteristics with it.

### 3.3. P. inopinatus THK-30 Effect on Teeth Bleaching with and without SHMP

**Figure 4:**
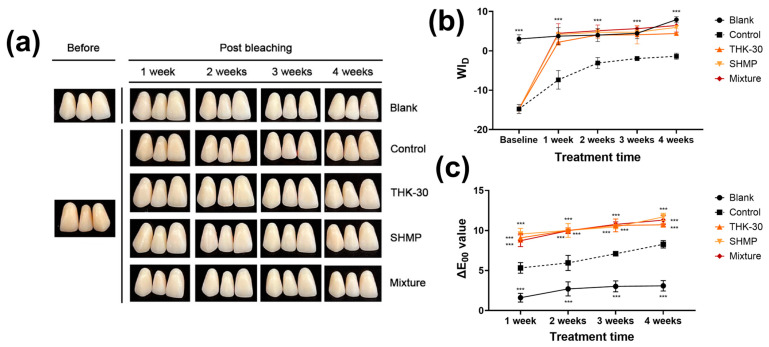
In-office bleaching treatment on stained artificial teeth. (**a**) Digital picture of result; (**b**) Whiteness Index for Dentistry of in-office bleaching treatment on artificial teeth; and (**c**) ∆E_00_ value of in-office bleaching treatment on artificial teeth. All experiments were performed at least three times, and the data are presented as mean ± standard deviation. *** *α* < 0.001 vs. control group.

**Figure 5:**
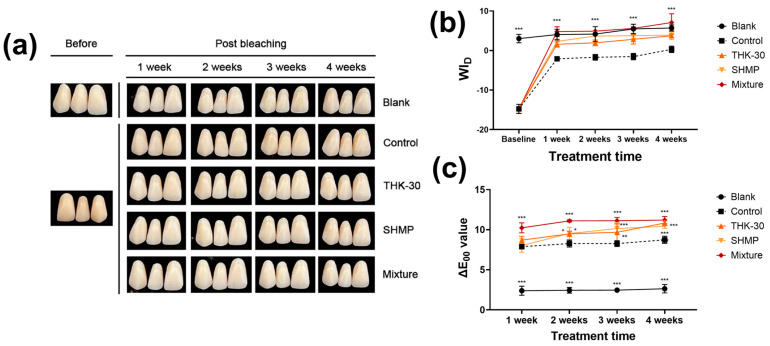
Brushing and mouthwash treatment on stained artificial teeth. (**a**) Digital picture of result; (**b**) Whiteness Index for Dentistry of brushing and mouthwash treatment on artificial teeth; and (**c**) ∆E_00_ value of brushing and mouthwash treatment on artificial teeth. All experiments were performed at least three times, and the data are presented as mean ± standard deviation. *** *α* < 0.001, ** *α* < 0.01, * *α* < 0.05 vs. control group.

The teeth bleaching effect test was performed in five distinct groups, including the control, THK-30 10 mg/mL treatment, SHMP 8% treatment, and 10 mg/mL THK-30, and 8% SHMP combination groups. The study conducted a comparative analysis of ΔL, Δa, Δb, and ΔE values between the baseline measurement and at 1, 2, 3, and 4 weeks following the application of a bleaching agent (Figure 4 and Figure 5, and Table 3). Regarding the L* values, the study observed a significant increase in all groups except for the blank group (Figure 4b and Figure 5b). The THK-30, SHMP, and mixture groups exhibited an upward trend in ΔE values from the first to the fourth week, while the control group displayed a decrease in ΔE values in the fourth week under the in-office bleaching conditions and in the second week under the brushing and mouthwash conditions (Figure 4c and Figure 5c).

The mixture group exhibited a more pronounced alteration in tooth color compared to the THK-30 and SHMP groups, regardless of whether brushing and mouthwash were employed or not. Under the in-office bleaching conditions, the mixture group demonstrated the most significant color change throughout the four-week experiment when compared to the other groups. Conversely, in the brushing and mouthwash conditions, the ΔE value for the mixture group was higher in the first and fourth weeks (Figure 4c and Figure 5c, and Table 3). The study observed an enhancement in brightness post-bleaching, while the Δa values continued to exhibit a greenish hue and Δb values retained their bluish characteristics (Table 3), indicating a distinct lack of discoloration. Notably, the obtained ΔE_00_ values indicate a substantial perceived change in tooth color, exceeding both the perceptibility threshold (PT) and acceptability threshold (AT) values determined in the study (PT at ΔE_00_ = 0.8 and AT at ΔE_00_ = 1.8 under simulated clinical conditions).

### 3.4. P. inopinatus THK-30 Synergy Effect in Antibacterial with SHMP

**Figure 6:**
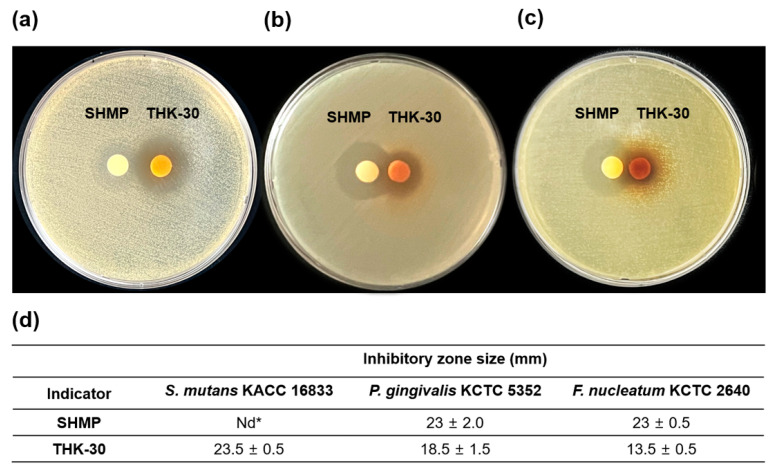
Synergy disc diffusion of *Pediococcus inopinatus* THK-30 and SHMP at 50 mg per disc against oral pathogens. Double-disc diffusion result of (**a**) *Streptococcus mutans* KACC 16833; (**b**) *Porphyromonas gingivalis* KCTC 5352; (**c**) *Fusobacterium nucleatum* KCTC 2640; and (**d**) detailed inhibition zone (mm) with bridge formation (positive result) for *Porphyromonas gingivalis* and *Fusobacterium nucleatum*. * (nd) non-detected.

The effects of THK-30 CFS and SHMP on three pathogenic strains, specifically *S. mutans* KACC 16833, *P. gingivalis* KCTC 5352, and *F. nucleatum* KCTC 2640, were assessed via double-disc diffusion assays. THK-30 CFS inhibits all three strains, whereas SHMP is more effective against Gram-negative bacteria. A synergy effect was also detected against Gram-negative stains between SHMP and THK-30 CFS, as illustrated by th bridges forming in between the inhibition rings of the two samples (Figure 6).

**Figure 7:**
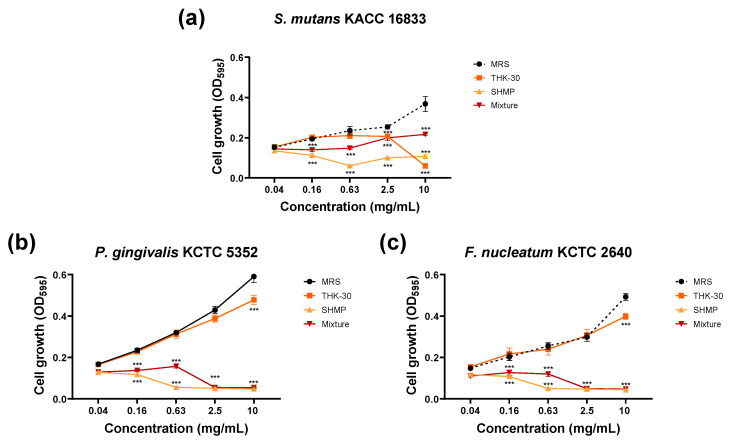
Antibacterial activity by CFS of *Pediococcus inopinatus* THK-30, SHMP, and their mixture against three oral pathogens related to halitosis: (**a**) *Streptococcus mutans* KACC 16833; (**b**) *Porphyromonas gingivalis* KCTC 5352; and (**c**) *Fusobacterium nucleatum* KCTC. All experiments were performed at least three times, and the data are presented as mean ± standard deviation. *** *p* < 0.001 vs. control group.

Further, both THK-30 CFS and SHMP exhibited noticeable impacts on all three strains, with a mild synergy observed against *P. gingivalis* KCTC 5352. In terms of MIC, SHMP displayed significant inhibition against Gram-negative pathogens, with a milder effect on *S. mutans* KACC 16833. When SHMP and THK-30 CFS were combined (referred to as the mixture group), the potent inhibitory effect against Gram-negative pathogens persisted, achieving an MIC of 1.25 mg/mL. This combined treatment proved markedly more effective than using THK-30 CFS alone, which had an MIC of 40 mg/mL against these pathogens. These findings highlight the synergistic potential of THK-30 and SHMP, particularly for combating Gram-negative oral pathogens (Figure 7).

### 3.5. P. inopinatus THK-30 Interferes with the Biofilm Formation of Oral Pathogen Bacteria

**Figure 8:**
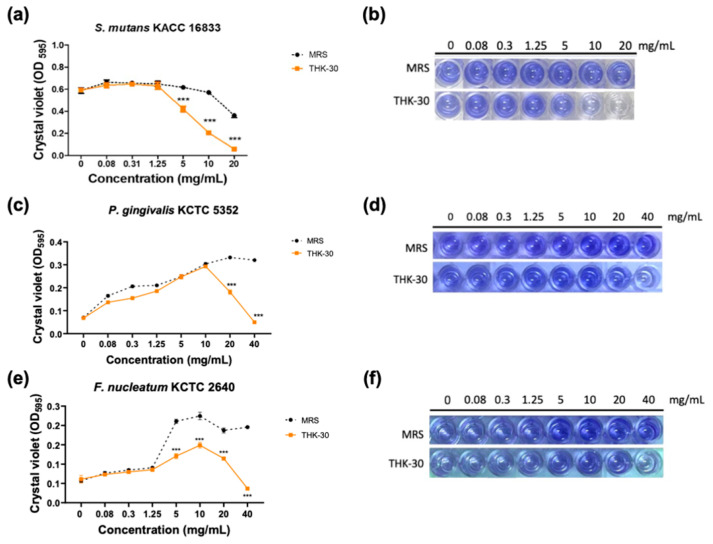
Antibiofilm formation activities of *Pediococcus inopinatus* THK-30. OD measurement against (**a**) *Streptococcus mutans* KACC 16833; (**c**) *Porphyromonas gingivalis* KCTC 5352; and (**e**) *Fusobacterium nucleatum* KCTC 2640. Representative images of a 96-well plate showcase the CV-stained biofilm of (**b**) *Streptococcus mutans* KACC 16833, (**d**) *Porphyromonas gingivalis* KCTC 5352, and (**f**) *Fusobacterium nucleatum* KCTC 2640. All experiments were performed at least three times, and the data are presented as mean ± standard deviation. *** *p* < 0.001 vs. control group.

This test showcases the findings of the inhibitory effect of biofilm formation against oral pathogens, including Gram-positive *S. mutans* KACC 16833 and Gram-negative *P. gingivalis* KCTC 5352 and *F. nucleatum* KCTC 2640. The control group demonstrated a slight increase and a dose-dependent decrease in biofilm formation for *S. mutans* KACC 16833 from treatment with 0.08 mg/mL MRS. Below the MIC concentration, the evaporated CFS of the THK-30 treatment group had no impact on the biofilm formation of the tested strains (Figure 8a,b). For Gram-negative pathogens, biofilm-forming inhibition started at the concentration of 10 mg/mL, with a dose-dependent effect (Figure 8c–f).

### 3.6. P. inopinatus THK-30 Interferes with the Artificial Teeth Biofilm Forming

**Figure 9:**
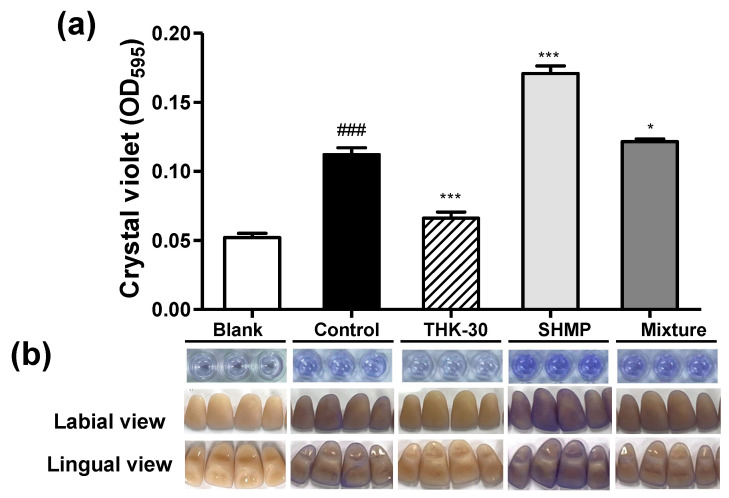
Antibiofilm formation activities of *Pediococcus inopinatus* THK-30, SHMP, and their mixture on artificial teeth. (**a**) The bar graph on teeth antibiofilm result; (**b**) representative images of a 96-well plate and treated artificial teeth demonstrating *Streptococcus mutans* biofilm, stained with CV, at 10 mg/mL THK-30 CFS and SHMP 8% concentration. All experiments were performed at least three times, and the data are presented as mean ± standard deviation. * *p* < 0.05, *** *p* < 0.001 compared to MRS control group using TSYS, and ^###^ *p* < 0.001 compared to blank group.

To explore the impact of THK-30 CFS and SHMP on *S. mutans* biofilm formation on artificial tooth surfaces, five groups were studied, including blank, control, THK-30 10 mg/mL, SHMP 8%, and the mixture of THK-30 treatment groups. The control group exhibited expected biofilm formation, while the THK-30 treatment demonstrated a significant inhibitory effect on *S. mutans* biofilm formation. Intriguingly, the SHMP treatment appeared to enhance biofilm formation despite showing an inhibitory effect in the MIC test, whereas the mixture group had a significantly lower level of biofilm formation compared to the SHMP group (Figure 9). These findings underscore the potential of THK-30, particularly in combination with SHMP, as a promising strategy to control *S. mutans* biofilm formation on artificial teeth, offering insights into the management of oral-biofilm-related concerns.

### 3.7. P. inopinatus THK-30 Active Substance Analysis Using Enzyme-Treating Assay

**Figure 10:**
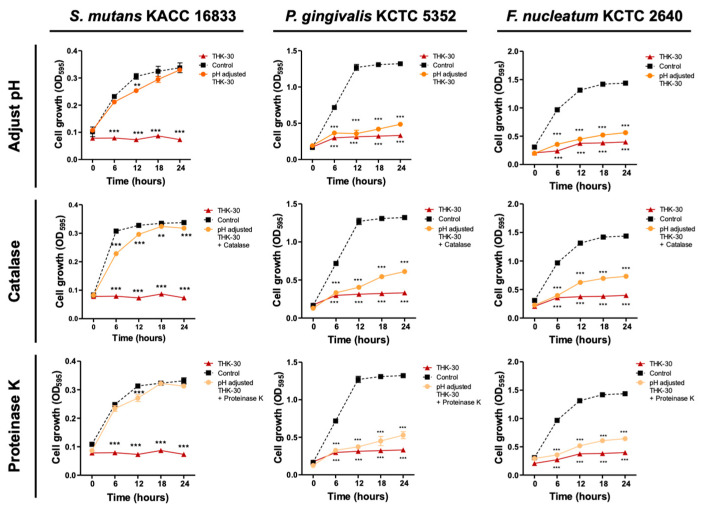
The inhibitory activity of THK-30 on oral pathogens after treatment with pH adjustment and enzymes. All experiments were performed at least three times, and the data are presented as mean ± standard deviation. ** *p* < 0.01 and *** *p* < 0.001 compared to MRS control group.

Notably, when Gram-positive *S. mutans* was subjected to treatment with pH-adjusted conditions and enzymes, the inhibition effect of THK-30 was nearly eradicated, with the pH level reaching that seen in the control group data. In contrast, when Gram-negative bacteria were treated with catalase and proteinase K enzymes, the inhibition effect of THK-30 was also diminished, although the decrease was not to the same extent as seen in *S. mutans* (Figure 10). Further investigation is required to elucidate the specific mechanisms underlying these observations.

## 4. Discussion

In this study, we pioneered the novel application of *P. inopinatus* THK-30 CFS, derived from kimchi, to bleach-stained artificial teeth. We found that doing so inhibited the growth of oral bacteria and prevented their biofilm formation.

**Figure 11:**
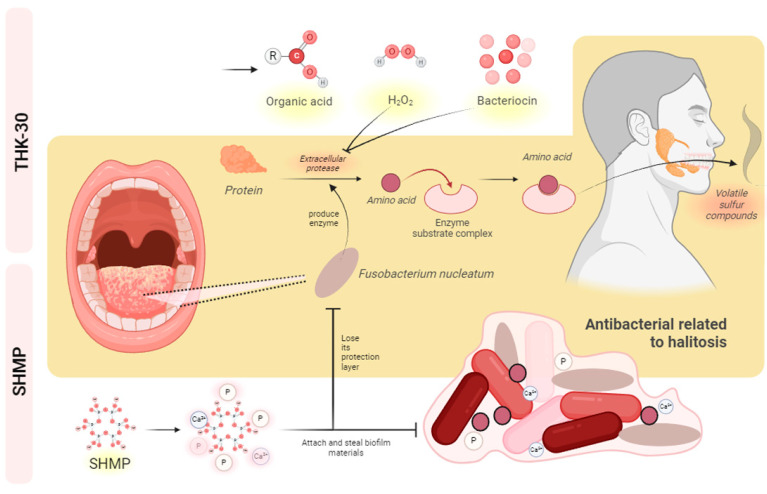
Antibacterial halitosis mechanism of THK-30 and SHMP. The figure was generated by BioRender.com.

When testing *P. inopinatus* THK-30 CFS for its antibacterial halitosis properties, the result showed that it effectively prevented oral pathogen growth and biofilm formation, either when used alone or combined with SHMP (Figure 2, Figure 6, Figure 7, Figure 8 and Figure 9). In line with previous studies utilizing crystal violet staining, SHMP, a natural absorbent, shows a stronger tendency to absorb the staining color. This indicates its ability to create a protective layer on the enamel surface, leading to a more vivid purple color of artificial teeth [35]. Meanwhile, our sample THK-30 demonstrated an effective reduction in this escalating purple staining condition (Figure 9). Specifically for halitosis-causing bacteria, THK-30 produces H_2_O_2_ and bacteriocins. In previous studies, this has shown the inhibition of *F. nucleatum*, with one of the papers being under review [36,37,38]. The *F. nucleatum*-inhibiting mechanism of THK-30 relies on H_2_O_2_ and bacteriocins to prevent the production of protease in *F. nucleatum* [39]. This effect in turn prevents amino acids from locking with their complex, preventing unpleasant VSC from being created [36,40]. Secondly, for the inhibition pathway, SHMP can capture biofilm components like calcium or phosphate of *F. nucleatum* and inhibit its survival (Figure 11). Significantly, SHMP plays a role in preventing dental calculus formation and accumulation by influencing mineral deposition in the oral environment [39]. Moreover, in a previous study, H_2_O_2_ and bacteriocins were found to not only directly inhibit a wide range of oral pathogens but also prevent the formation and development of biofilms, commonly referred to as calculus in dental care, by interfering with microbial communication known as quorum sensing [41]. This interference makes it more challenging for *F. nucleatum* to form biofilms in the context of the oral environment.

The causes of halitosis are intricate, involving various factors, including dental caries, periodontal disease, oral microbiome imbalance, and psychological conditions [42]. In this study, bacterial inhibition by THK-30 alleviated intraoral halitosis related to the bacteria causing halitosis. However, further study on the sample effect with other halitosis etiologies, such as psychological factors or an unbalanced gastrointestinal tract [28], is required for the future application of THK-30. THK-30 also inhibits important oral pathogens, including *S. mutans*, which is crucial in dental plaque and other oral conditions, by producing organic acids (Figure 10). THK-30 organic acids are safe from acidity-causing dental caries [9]. Any excessive acid will naturally be neutralized by saliva, which is confirmed by previous studies [9,43,44]. Importantly, live probiotics like the THK-30 strain can compete with cariogenic bacteria and inhibit tooth decay [45,46]. These results suggest the safe use of LAB strain THK-30 for addressing bacterial-related halitosis and other potential issues with dental caries bacteria. Additionally, previous studies on probiotics-based products, like toothpaste and chewing-gum, have shown their efficacy in inhibiting periodontitis [47], further supporting the exploration of these interventions for the development of effective halitosis prevention strategies that tackle its various causes [42] using probiotic-based oral care products.

Regarding teeth bleaching, *P. inopinatus* THK-30 CFS, whether used alone or in combination with SHMP, showcases significant teeth-bleaching capacity. The discolored artificial teeth appeared to whiten noticeably when observed with the naked eye (Figure 4 and Figure 5). This change was also confirmed through a detailed analysis using measurement systems called CIEDE2000 and WI_D_ and occurred from the first week of treatment for both conditions. This effect was maintained with no significant change during the three following weeks (Figure 4 and Figure 5). The result of THK-30 use was significantly improved and faster than that using 3% H_2_O_2_, which only showed effectiveness from the second week of treatment in previous studies [48].

**Figure 12:**
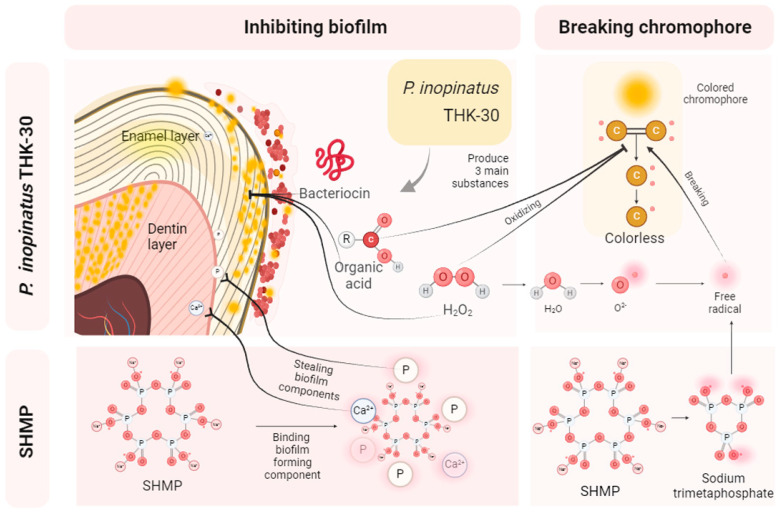
Indirect bleaching by anti-biofilm and direct bleaching mechanisms of THK-30 and SHMP. The figure was generated by BioRender.com.

There are two main mechanisms responsible for tooth bleaching. One mechanism involves the direct oxidation of double-bond chromophores in dietary compounds by H_2_O_2_ and organic acid produced as a result of THK-30 treatment, which is typically generated by LAB [49,50]. The second is the suppression of biofilm formation by entangling those color-bearing molecules, facilitating teeth bleaching via the production of organic acids, H_2_O_2_, and bacteriocins from THK-30. Previous in-depth analysis revealed that double carbon bonds are responsible for the yellow color of stains due to light absorption. These bonds transform into hydroxyl groups, similar to colorless alcohol, leading to the loss of the yellow hue in the stain molecules, as demonstrated in Figure 12 [51]. For the bleaching teeth effect, SHMP combined with H_2_O_2_ produced by THK-30 released a higher number of free radicals and enhanced their teeth-bleaching result. In a previous study by Dr. Shobana, using probiotic pre-treatment on bleached enamel demonstrated promising potential to enhance the bond between composite resin and enamel, possibly contributing to the overall strength and integrity of the teeth via bleaching treatment [52]. This finding suggests a potential alternative for conventional bleaching materials, with fewer drawbacks such as transient tooth sensitivity [53], gum irritation, and enamel erosion associated with high-concentration acidic bleaching gels [54].

Furthermore, H_2_O_2_ stability can be improved with the addition of SHMP to preserve other proteins [55]. The experiment’s results propose the application of methods including the use of gel with silicone molds for at-home or in-office bleaching, toothpaste, and mouthwash. The combination of THK-30 and SHMP will offer a more stable and increased effectiveness in teeth bleaching and the treatment of intraoral halitosis. However, additional research is necessary to comprehend their mechanisms of action and also assess their effectiveness and safety in clinical settings.

Our study exploring the use of *P. inopinatus* THK-30 CFS from kimchi for teeth bleaching and bacterial inhibition has certain limitations. The use of resin teeth was decided on due to practical constraints in obtaining real teeth and the versatility of resin teeth in simulating dental scenarios. While our findings show promise in inhibiting oral pathogens and bleaching teeth, further research is needed to understand mechanisms, safety, and effectiveness in diverse clinical settings, particularly in addressing non-bacterial causes of halitosis.

## 5. Conclusions

In conclusion, our investigation into the potential of *P. inopinatus* THK-30 from kimchi for use in teeth bleaching and to address oral bacterial infections leading to halitosis aligns with our initial objective. The study results support our hypothesis, indicating that the exploration of dental applications for *P. inopinatus* THK-30 holds promise for advancing safe and effective treatment in the domains of dental aesthetics and oral health. In the future, *P. inopinatus* THK-30 is expected to hold potential as not only a standalone material but also in combination with other functional ingredients in the development of oral functional materials, with futher clinical study needed on its effectiveness.

## Figures and Tables

**Table 1:** The minimum inhibition concentration (MIC) and minimal bactericidal concentration (MBC) of *Pediococcus inopinatus* THK-30 CFS against oral pathogenic bacteria are depicted. All experiments were performed at least three times, and the data are presented as mean ± standard deviation.

Strain	MIC (mg/mL)	MBC (mg/mL)
Gram-positive bacteria		
*Streptococcus mutans* KACC 16833	≥5	10
*Streptococcus gordonii* KACC 13829	≥2.5	5
*Streptococcus mitis* KACC 16832	≥2.5	5
*Streptococcus downei* KACC 13827	≥5	nd ^1^
*Streptococcus ferus* KACC 13881	≥1.25	2.5
Gram-negative bacteria		
*Porphyromonas gingivalis* KCTC 5352	40	nd
*Fusobacterium nucleatum* KCTC 2640	40	nd

^1^ (nd) non-detected.

**Table 2:** Cultural characteristics of *Pediococcus inopinatus* THK-30 and DSM 20285^T^ and their carbon source utilization.

	THK-30	DSM 20285^T^
Isolation Source:	Kimchi	Brewery Yeast
Growth Temperature:		
25 °C	+ ^1^	+
30 °C	+	+
37 °C	w ^2^	w
45 °C	w	w
Acid produced from:		
D-galactose	+	+
D-glucose	+	+
D-fructose	+	+
D-mannose	+	+
Methyl-α-D-mannopyranoside	− ^3^	−
N-acetylglucosamine	+	+
Amygdalin	+	+
Esculin ferric citrate	+	+
Salicin	+	+
D-cellobiose	+	+
D-maltose	w	w
D-trehalose	+	+
Gentiobiose	+	+
D-turanose	−	−

^1^ (+) positive; ^2^ (w) weakly positive; ^3^ (−) negative.

**Table 3:** ΔL*, Δa*, and Δb* value of teeth bleaching treatment from one to four weeks of treatment.

Treatment Time (Week)	Group	In-Office Bleaching						Brushing and Mouthwash					
		ΔL		Δa		Δb		ΔL		Δa		Δb	
		Mean	SD	Mean	SD	Mean	SD	Mean	SD	Mean	SD	Mean	SD
1	Blank	−1.233	0.513	0.100	0.000	1.367	0.306	−1.667	0.208	−0.633	1.877	−2.133	0.850
	Control	−0.033	0.404	−2.067	0.416	−5.967	2.003	0.667	0.208	−2.700	0.100	−7.067	1.858
	THK−30	3.033	0.153	−2.867	0.379	−7.733	1.940	0.967	0.153	−2.733	0.115	−6.933	2.013
	SHMP	3.400	0.721	−3.500	0.100	−7.967	1.620	3.367	0.058	−2.433	0.153	−7.433	1.943
	Mix	3.933	0.586	−3.367	0.058	−9.567	1.893	4.300	0.000	−2.833	0.208	−9.700	1.992
2	Blank	−2.333	0.153	0.133	1.531	−3.767	0.462	−2.600	0.265	−0.400	0.100	−3.333	0.231
	Control	0.000	0.436	−2.400	0.100	−7.133	1.358	2.100	0.100	−2.267	0.058	−7.067	1.629
	THK−30	3.233	0.058	−3.467	0.252	−7.267	1.332	2.967	0.115	−3.067	0.115	−7.400	1.778
	SHMP	3.933	0.115	−2.900	0.361	−8.833	0.907	4.100	0.300	−2.767	0.058	−8.700	1.400
	Mix	4.267	0.153	−3.200	0.265	−9.000	0.800	4.333	0.153	−2.700	0.200	−6.333	1.290
3	Blank	−2.233	0.306	−0.400	0.346	−0.733	0.404	−3.367	0.252	−0.567	0.153	−0.733	0.153
	Control	1.233	0.379	−2.667	0.058	−8.800	1.992	1.967	0.379	−1.600	0.100	−4.867	1.629
	THK−30	3.133	0.611	−2.400	0.000	−3.600	1.836	5.133	0.153	−3.033	0.153	−9.100	1.833
	SHMP	5.933	0.252	−2.633	0.115	−7.400	2.821	4.567	0.231	−2.367	0.115	−9.467	2.053
	Mix	4.233	0.058	−3.233	0.058	−8.200	1.836	5.167	0.153	−2.500	0.265	−7.267	3.202
4	Blank	−2.200	0.624	−1.100	0.265	−2.533	0.503	−0.533	0.493	−0.067	0.321	−1.600	1.735
	Control	3.067	0.493	−1.633	0.153	−6.467	2.857	2.900	0.400	−0.633	0.115	−0.800	1.442
	THK−30	3.767	0.058	−2.933	0.153	−8.600	1.873	5.767	0.577	−2.233	0.115	−8.867	2.026
	SHMP	5.233	0.115	−3.067	0.058	−9.433	1.484	5.833	0.404	−2.067	0.153	−7.933	1.747
	Mix	6.000	0.361	−2.933	0.058	−8.133	1.677	5.300	0.529	−2.900	0.300	−9.367	2.122

## Data Availability

Data can be made available upon request.
